# Angioedema after ingestion of persimmon fruit

**DOI:** 10.1186/2045-7022-3-S3-P156

**Published:** 2013-07-25

**Authors:** E Shehu, M Hoxha, D Qama, A Priftanji

**Affiliations:** 1 Internal Medicine, Allergy and Clinical Immunology, Regional Hospital of Durres, Durres, Albania; 2 Allergy and Clinical Immunology, UHC *Mother Teresa*, Tirane, Albania; 3 Allergy and Clinical Immunology, Regional Hospital of Berat, Berat, Albania

## Background

The Persimmon tree is native to Far East, where it has been cultivated for centuries. Diospyros kaki belongs to the Ebenaceae family. These fruits are high in glucose and protein, and also have various medicinal and chemical uses.

## Methods

A 41-year-old woman developed severe facial angioedema and dyspnea within 15 minutes of persimmon fruit intake. She was treated with intravenous corticosteroids and antihistamines, monitored in a hospital for two days and attended our clinic the next week for assessment of possible fruit allergy. She reported a previous episode of angioedema and generalized urticaria some months ago, immediately after had eaten a kaki. The patient had an allergic rhinitis to house dust mites and also she suffered diabetes mellitus. She was in treatment with metformine 1500 mg a day. Skin prick tests were positive for Dermatophagoides pteronyssinus and farinae. (Stallergenes, Cedex, France). Prick by prick were performed by placing a piece of persimmon fruit on the forearm and pricking through them and itwas positive in our patient (++++) but not in 3 healthy controls. We realized also an open food challenge with fresh persimmon fruit. Traces of this fruit were introduced to the patient initially by contacting the lip without getting any allergic symptoms. Finally a substantial amount of the fruit was consumed and 15 minutes later our patient experienced pruritus, facial angioedema and dyspnea. The test was stopped when the adverse reaction occured.

## Results

There are only a few case reports of allergic reaction to persimmon fruit in the literature. There is only one report of an anaphylactic reaction caused by persimmon fruit ingestion in a patient sensitized to dust mites.

## Conclusion

In the patient presented, we found a positive skin prick test in response to persimmon fruit. Accordingly, we instructed the patient to avoid persimmon fruit. She received rescue medications for self-treatment in case of an emergency. Specific IgE for persimmon fruit are not available in our country.In our case, an IgE mediated mechanism was supported by inmediate positive skin reaction.

## Disclosure of interest

E Shehu: Consultant for GSK, Albania, M Hoxha: None declared, D Qama: None declared, A Priftanji: None declared.

